# Reliability, validity, and cross-cultural adaptation study of the Turkish version of the Patient-Rated Wrist/Hand Evaluation questionnaire

**DOI:** 10.3906/sag-1806-37

**Published:** 2019-04-18

**Authors:** Deniz ÖKE TOPCU, Sevği İKBALİ AFŞAR

**Affiliations:** 1 Department of Physical Medicine and Rehabilitation, Eyüpsultan State Hospital, İstanbul Turkey; 2 Department of Physical Medicine and Rehabilitation, Faculty of Medicine, Başkent University, Ankara Turkey

**Keywords:** Patient-Rated Wrist/Hand Evaluation, cross-cultural adaptation, validity, reliability, Turkish version

## Abstract

**Background/aim:**

The aim of this study was to cross-culturally adapt the Turkish version of the Patient-Rated Wrist/Hand Evaluation (T-PRWHE) questionnaire for use in the Turkish patient population. Moreover, we aimed to evaluate the reliability and validity of the T-PRWHE questionnaire.

**Materials and methods:**

A total of 166 patients with hand and wrist problems were included in the study. They completed the T-PRWHE, the Disabilities of the Arm, Shoulder, and Hand (DASH) questionnaire, and the 36-Item Short-Form Health Survey (SF-36) at baseline and at the 3rd month of the study. Reliability was evaluated by analyzing internal consistency (Cronbach alpha coefficient) and test-retest reliability (intraclass correlation coefficient). To analyze validity, factor analysis of the T-PRWHE and correlation coefficients between the T-PRWHE, DASH, and SF-36 were obtained.

**Results:**

Reliability of the T-PRWHE in terms of internal consistency (Cronbach alpha coefficients for T-PRWHE were found to be 0.85) was excellent. Intraclass correlation coefficients were over 0.90. The T-PRWHE has three factors and the correlations between the T-PRWHE and DASH and SF-36 were statistically significant.

**Conclusion:**

Based on the results obtained, the Turkish version of the PRWHE questionnaire was found to be a valid and reliable scale and it is recommended for the evaluation of patient-based pain and disability level in routine clinical practice.

## 1. Introduction

The traditional methods for evaluating hand and wrist function following an intervention consist of measuring grip strength and assessing the range of motion, which both provide an objective analysis of the outcomes. However, these methods are insufficient in determining the dependence level of the patient in daily activities and revealing the performance of daily living activities from the patient’s own point of view [1]. 

In recent decades, questionnaires that are specific to the region where the pathology is located and that evaluate the functional status and disability level of the patient suffering from musculoskeletal system disorders have been developed [2]. The Disabilities of the Arm, Shoulder, and Hand (DASH) questionnaire is one of the most important self-report tools for the upper extremities. The Patient-Rated Wrist Evaluation (PRWE) questionnaire was designed in order to specifically evaluate function after wrist injuries as the DASH questionnaire concerns all upper extremities [3,4]. The PRWE questionnaire, which was developed to measure wrist pain and disability in daily living activities of patients with distal radius fracture, has been shown to be suitable for usage in many pathologies concerning the wrist and to have perfect reliability, validity, and responsiveness [1,4,5]. MacDermid et al. reported the PRWE questionnaire to be more sensitive than the DASH and 36-Item Short-form Health Survey (SF-36) questionnaires in the evaluation of patients with wrist injury [6]. The most important advantage of this questionnaire is that it is short and easy to complete. The validity, reliability, and responsiveness of the PRWE have therefore been tested in many different populations [4,7–13]. The PRWE questionnaire was modified to evaluate problems in the hand together with the wrist and was named the Patient-Rated Wrist and Hand Evaluation (PRWHE). It was found to be a valid and reliable questionnaire in various clinical situations regarding the hand and wrist.****The wrist problems comprised wrist fractures, carpal instabilities, and osteoarthritis. The most common diagnoses in patients with hand problems were hand fractures, tendon lacerations, palmar fasciectomy, or finger joint arthroplasty (metacarpophalangeal joint or proximal interphalangeal joint) [5]. The PRWHE questionnaire has been translated into Arabic, Italian, and Dutch and cross-cultural adaptation studies have been conducted [14–16]. 

The aim of this study is to cross-culturally adapt the PRWHE questionnaire for use in the Turkish population and to test the reliability, test-retest reliability, and validity in a group of patients having pathologies of the hand and wrist.

## 2. Materials and methods

### 2.1. Patients

One hundred and sixty-six patients with orthopedic wrist and hand injuries, treated by surgical or conservative methods and referred to the physical therapy and rehabilitation clinic, were prospectively recruited for the study.

The inclusion criteria were the presence of pain symptoms due to various types of hand and/or wrist injury, age over 18 years, native speaker of Turkish, and able to complete the questionnaire without help. Patients with hand/wrist injury originating from rheumatologic and/or neurological disorders and patients with pathologies associated with other upper extremity joints were excluded from the study.

This study was approved by the university’s institutional review board and ethics committee (Project no: KA12/254). All subjects understood the purpose of the study and provided their written informed consent prior to their participation in the study.

### 2.2. Outcome measures

#### 2.2.1. Patient-Rated Wrist and Hand Evaluation Questionnaire 

The PRWHE is a 15-item questionnaire designed to measure pain and function of the wrist and hand joints [4]. It consists of two subscales: a pain subscale (PRWHE-P) and a functional subscale (PRWHE-F). The PRWHE-P consists of five items on the severity and frequency of the pain. The PRWHE-F is divided into two subsections; the specific function (PRWHE-SF) subscale has six items and the usual function (PRWHE-UF) subscale has four items. Each item is scored on a 0–10 scale. The total score is achieved by adding the PRWHE-P score (sum of the first 5 items) to the PRWHE-F score (sum of the 10 items divided by two). Thus, the pain and function scores carry equal weight in the scoring system. A score closer to 0 indicates less pain and lower disability level while a score closer to 100 indicates more pain and disability. There is also an optional appearance (PRWHE-A) section. The patient evaluates the importance of the hand’s appearance as very important, a little important, or not important at all and the disturbance felt from the appearance is graded between 0 and 10 [5]. The score from the appearance section that is answered voluntarily is not included in the total score.

#### 2.2.2. Disabilities of the Arm, Shoulder, and Hand (DASH) Questionnaire

This questionnaire was developed for evaluation of the entire or partial upper extremity in patients with an upper extremity problem. It includes 21 physical functional items, 6 symptom items, and 3 social/role function items. Each item has 5 possible answers and the total score is between 0 and 100. Higher scores indicate the worst perceptions of pain and disability of the upper limb. The Turkish validity and reliability study of this questionnaire was conducted in 2006 [17]. It was shown to be useful in the evaluation of shoulder, elbow, and wrist injuries [1,18].

#### 2.2.3. The 36-Item Short-Form Health Survey

The SF-36 is a self-report quality of life assessment questionnaire. It reflects subjective feedback on the physical condition of the patient. It is divided into eight subscales: physical functioning (PF), role limitations due to physical health problems (RP), bodily pain (BP), general health perceptions (GH), vitality (VT), social functioning (SF), role limitations due to emotional problems (RE), and mental health (MH). Each scale is scored in a range from 0 to 100 with a score of 0 showing poor health status and 100 showing good health status [19]. The SF-36 is considered a reliable, valid, and responsive tool and it has been tested in several populations, including a Turkish population [20].

### 2.3. Clinical evaluation protocol 

The age, sex, affected and dominant extremities, diagnosis, and treatment methods of the patients included in the study were recorded. All patients completed the following questionnaires at baseline and at the 3rd month of follow-up: T-PRWHE, DASH, and SF-36. In a group of 36 patients, the T-PRWHE was administered a second time 7–10 days after the initial assessment for the test-retest reliability. The interval of 7–10 days was chosen because it was unlikely that the patient’s condition would substantially change. However, the time interval would be large enough for the patients to forget their first answers to the questions.

### 2.4. Translation and cultural adaptation process

The PRWHE’s translation from English into Turkish was done following the guidelines published by Beaton et al. [21]. Permission for the development of the Turkish version was received by e-mail from Joy MacDermid, the author who developed the original scale. 

The English original of the scale was translated to Turkish by two independent bilingual translators who were native speakers of Turkish and were trained in medicine. The translations were compared and the Turkish text was created from the statements that best represented each item. The text obtained was later translated again into English by two professional translators with English as their native language who were independent of the study. The text obtained was compared with the original PRWHE questionnaire. The Turkish text was evaluated and the requirement for cultural adaptation was determined by a team consisting of translators with English as their native language, an English linguist, a physiatrist, a family physician, and a pharmacology specialist. Based on the results, minor changes were made in the Turkish adaptation of the scale. “Pound” is not used in Turkey as a measurement unit and was changed to “kilogram”. After it was concluded that both versions were consistent with each other, the Turkish form was finalized (Appendix 1). A pilot study was then conducted on 30 subjects who were literate and diagnosed with a disorder related to the hand and wrist. The aim of the pilot study was to determine any unclear aspect not understood by the patients in the questionnaire. The cultural adaptation study was concluded with the determination of equivalence between the Turkish adaptation and the English original.

### 2.5. Statistical analysis

The Kolmogorov–Smirnov test was used to evaluate normal distribution of data. Continuous variables were expressed as mean ± standard deviation and median (minimum–maximum). Categorical variables were presented as numbers and percentages. The paired t-test or Wilcoxon test was used to determine whether T-PRWHE, DASH, and SF-36 scores at follow-up showed a significant improvement versus the baseline. 

Reliability represents the ability of an instrument to yield consistent and reproducible results. In the present study, reliability was evaluated by analyzing internal consistency and test-retest reliability. The internal consistency was estimated using Cronbach’s alpha (CA) coefﬁcient [22], and a value of >0.7 was considered to indicate satisfactory internal consistency [23]. Test-retest reliability of the T-PRWHE and its subscales was assessed by obtaining the intraclass correlation coefﬁcients (ICC) [24]. Test-retest reliability was considered acceptable for ICC values of >0.75 [25].

Criterion validity was assessed by Pearson correlation testing the predefined hypothesis concerning the expected relationship between the T-PRWHE and DASH scores and T-PRWHE and SF-36 scores. 

In order to determine the construct validity of the T-PRWHE questionnaire, factor analysis (principal components extraction with varimax rotation, eigenvalues of >1) was performed using the subscales of the self-report measure as the following items: PRWHE-P, PRWHE-UF, and PRWHE-SF.

Marginal homogeneity testing was used to determine whether PRWHE-A scores at follow-up showed a significant improvement versus the baseline. 

All statistics were extracted from SPSS 20.0. The critical values for signiﬁcance were set at P < 0.05. 

## 3. Results

A total of 166 patients, 93 men and 73 women, were included in the study. The diagnoses and demographic characteristics of the patients are presented in Table 1. 

**Table 1:** Demographic and clinical characteristics of the patients.

Variables		Results
Age, years (mean ± standard deviation)		45.8 ± 1.3
Sex	Female	73 (44%)
	Male	93 (56%)
Hand dominance	Right	147 (88.6%)
	Left	19 (11.4%)
Affected side	Right	115 (69.3%)
	Left	43 (25.9%)
	Bilateral	8 (4.8%)
Treatment method	Surgical	90 (54.2%)
	Conservative	76 (45.8%)
Diagnosis	Distal radius fracture	102 (61.5%)
	Distal radius and ulna fracture	13 (7.8%)
	Scaphoid fracture	12 (7.2%)
	Ganglion	8 (4.8%)
	De Quervain’s tenosynovitis	7 (4.2%)
	Metacarpal fracture	6 (3.6%)
	Trigger finger	4 (2.4%)
	Carpal lesions	4 (2.4%)
	Ligament injury	4 (2.4%)
	Triangular fibrocartilage complex injury	2 (1.2%)
	Tendinitis	2 (1.2%)
	Proximal phalanx fracture	2 (1.2%)

The T-PRWHE, DASH, and SF-36 scores of the patients at baseline and the 3rd month are presented in Table 2. A significant decrease in the T-PRWHE and DASH scores (P < 0.05) and a significant increase in the SF-36 scores (P < 0.05) were found. T-PRWHE score differences at follow-up compared to the baseline values (ΔT-PRWHE = T-PRWHE1 – T-PRWHE2) showed a minimum decrease of 21 points and maximum improvement of 95 points, with a median value of 55 points. The CA coefficient calculated for the evaluation of internal consistency was found to be 0.85 for the T-PRWHE total score. The CA coefficients for the pain and function subscales were 0.79 and 0.92, respectively. This analysis showed that the T-PRWHE total and both subscales had excellent internal consistency (Table 2). In addition, the ICC for the total T-PRWHE and its subscales demonstrated excellent test-retest reliability (Table 2). 

**Table 2:** Scores for the T-PRWHE, DASH, and SF-36 questionnaires at baseline and follow-up.

Variables	Baseline		Follow-up		P	Test-retest reliability			Cronbach α	Cronbach α range
	Mean ± SD	Median (min–max)	Mean ± SD	Median(min–max)		ICC	95% CI	P*		
PRWHE-P	31.1 ± 10.2	32 (0–50)	8.7 ± 9.7	6.5 (0–50)	<0.001 α	0.991	0.982–0.995	<0.001	0.79	0.75–0.81
PRWHE-SF	39.4 ± 16.4	39.5 (0–60)	7.6 ± 8.6	5 (0–43)	<0.001 α	0.988	0.976–0.994	<0.001	0.80	0.77–0.82
PRWHE-UF	23.3 ± 10.9	24 (0–40)	4.6 ± 5.6	3 (0–28)	<0.001α	0.976	0.948–0.988	<0.001	0.82	0.80–0.82
PRWHE-F	31.3 ± 12.6	31.5 (0–50)	6.1 ± 6.9	4 (0–36)	<0.001 α	0.988	0.976–0.994	<0.001	0.92	0.89–0.92
T-PRWHE	62.4 ± 20.2	66.3 (0–99)	14.8 ± 16.0	11.3 (0–85)	<0.001α	0.994	0.988–0.997	<0.001	0.85	0.82–0.91
DASH-S	56.6 ± 20.4	55.4 (0–100)	14.8 ± 15.5	10 (0–77.5)	<0.001 α	0.966	0.934–0.982	<0.001	-	-
DASH-W	56.6 ± 20.4	56.3 (0–100)	17.5 ± 22.1	12.5 (0–88)	<0.001 α	-	-	-	-	-
SF-PF	64.2 ± 21.5	65 (0–100)	80.6 ± 18.2	85 (15–100)	<0.001β	0.972	0.947–0.986	<0.001	-	-
SF-RP	39.3 ± 39.7	25 (0–100)	72.9 ± 37.0	100 (0–100)	<0.001 α	0.962	0.928–0.981	<0.001	-	-
SF-BP	42.7 ± 20.0	41 (0–90)	72.8 ± 19.0	84 (21–90)	<0.001 α	0.811	0.631–0.904	<0.001	-	-
SF-GH	58.2 ± 19.5	57 (5–100)	62.7 ± 19.6	67 (20–97)	<0.001 α	0.794	0.633–0.889	<0.001	-	-
SF-VT	54.4 ± 17.3	55 (0–95)	57.2 ± 18.7	60 (5–95)	0.009β	0.909	0.828–0.952	<0.001	-	-
SF-RE	47.8 ± 32.5	33.3 (0–100)	86.9 ± 28.6	100 (0–100)	<0.001 α	0.872	0.765–0.932	<0.001	-	-
SF-MH	62.8 ± 17.5	64 (4–100)	72.1 ± 14.2	76 (20–100)	<0.001β	0.829	0.691–0.909	<0.001	-	-
SF	56.4 ± 21.8	50 (0–100)	90.7 ± 16.2	100 (25–100)	<0.001 α	0.758	0.628–0.892	<0.001	-	-

α: Baseline vs. follow-up (Wilcoxon test was used); β: baseline vs. follow-up (paired t-test was used); *: intraclass correlation significance (Pearson analysis) T-PRWHE: Turkish version of the Patient Rated Wrist/Hand Evaluation; PRWHE-P: pain subscale of the T-PRWHE; PRWHE-SF: specific functional subscale of the T-PRWHE; PRWHE-UF: usual Functional subscale of the T-PRWHE; PRWHE-F: functional subscale of the T-PRWHE; PRWHE-A: appearance subscale of the T-PRWHE, DASH-S: symptom subscale of the Disabilities of the Arm, Shoulder, and Hand Scale, DASH-W: work subscale of the DASH; SF-PF: physical functioning subscale of the 36-Item Short-Form Health Survey; RP: role physical; BP: bodily pain; GH: general health; VT: vitality; S: social functioning; RE: role emotional; MH: mental health.

The validity was evaluated as criterion-related validity and construct validity. The correlation of the T-PRWHE with the DASH and SF-36 was investigated for criterion-related validity. 

At the beginning, the correlation between T-PRWHE and DASH-symptom subscale (DASH-S) scores was statistically significant (P < 0.01), but there was no statistically significant correlation with T-PRWHE and the DASH-work subscale (DASH-W) (P > 0.05). There was a low correlation between PRWHE-P and some subscales of the SF-36 (physical functioning, role physical, bodily pain, general health, and viability), but no statistically significant relationship was found with other subgroups of the SF-36 (social functioning, role emotional, mental health) (Table 3).

**Table 3:** Correlations between T-PRWHE, DASH, and SF-36 at baseline.

	PRWHE-P	PRWHE-SF	PRWHE-UF	PRWHE-F	T-PRWHE	PRWHE-A
PRWHE-P	-	0.521*	0.510*	0.558*	0.855*	0.050
PRWHE-SF	0.521*	-	0.700*	0.952*	0.858*	0.196**
PRWHE-UF	0.510*	0.700*	-	0.885*	0.813*	0.206*
RWHE-F	0.558*	0.952*	0.885*	-	0.908*	0.215*
T-PRWHE	0.855*	0.858*	0.813*	0.908*	-	0.160**
PRWHE-A	0.050	0.196**	0.206*	0.215	0.160**	-
DASH-S	0.450*	0.636*	0.653*	0.694*	0.663*	0.152
DASH-W	0.232	0.440*	0.390*	0.481*	0.462*	0.017
SF-PF	–0.254*	–0.292*	–0.323*	–0.328*	0.335*	0.023
SF-RP	–0.193**	–0.183**	–0.170**	–0.192**	0.218*	0.207*
SF-BP	–0.453*	–0.268*	–0.339*	–0.319*	0.430*	0.050
SF-GH	–0.267*	–0.249*	–0.271*	–0.280*	0.309*	0.256*
SF-VT	–0.161**	–0.131	–0.138	–0.145	0.173*	0.059
SF-RE	–0.086	–0.058	–0.081	–0.072	0.090	0.015
SF-MH	–0.090	–0.167**	–0.159**	–0.178**	0.158**	0.302*
SF	–0.144	–0.344*	–0.387*	–0.390*	0.316*	0.228*

*P < 0.01, **P < 0.05 (Spearman correlation analysis was used). T-PRWHE: Turkish version of the Patient Rated Wrist/Hand Evaluation; PRWHE-P: pain subscale of the T-PRWHE; PRWHE-SF: specific functional subscale of the T-PRWHE; PRWHE-UF: usual functional subscale of the T-PRWHE; PRWHE-F: functional subscale of the T-PRWHE; PRWHE-A: appearance subscale of the T-PRWHE; DASH-S: symptom subscale of the Disabilities of the Arm, Shoulder, and Hand Scale; DASH-W: work subscale of the DASH; SF-PF: physical functioning subscale of the 36-Item Short-Form Health Survey (SF-36); RP: role physical; BP: bodily pain; GH: general health; VT: vitality; SF: social functioning; RE: role emotional; MH: mental health.

At the 3rd month, the correlation between T-PRWHE and DASH-S scores was statistically significant (P < 0.01). When subgroups of the T-PRWHE were examined, there was a strong correlation between PRWHE-P and DASH-S, while there was a moderate correlation between PRWHE-P and DASH-W scores (P < 0.01). A moderate correlation was also found between the PRWHE-P and subscales of the SF-36 (P < 0.01). The correlation results of the questionnaire scores applied at the beginning and at the 3rd month are presented in Table 4.

**Table 4:** Correlations between T-PRWHE, DASH, and SF-36 at 3 months of follow-up.

	PRWHE-P	PRWHE-SF	PRWHE-UF	PRWHE-F	T-PRWHE	PRWHE-A
PRWHE-P	-	0.846*	0.810*	0.850*	0.974*	0.247*
PRWHE-SF	0.846*	-	0.908*	0.985*	0.939*	0.316*
PRWHE-UF	0.810*	0.908*	-	0.965*	0.908*	0.251*
PRWHE-F	0.850*	0.985*	0.965*	-	0.947*	0.301*
T-PRWHE	0.974*	0.939*	0.908*	0.947*	-	0.280*
PRWHE-A	0.247*	0.316*	0.251*	0.301*	0.280*	-
DASH-S	0.790*	0.823*	0.791*	0.829*	0.837*	0.354*
DASH-W	0.681*	0.833*	0.823*	0.843*	0.786*	0.422*
SF-PF	0.430*	0.488*	0.480*	0.497*	0.475*	0.108
SF-RP	0.579*	0.625*	0.604*	0.634*	0.623*	0.256*
SF-BP	0.729*	0.700*	0.688*	0.712*	0.748*	0.278*
SF-GH	0.245*	0.262*	0.256*	0.264*	0.263*	0.078
SF-VT	0.352*	0.344*	0.318*	0.339*	363*	0.094
SF-RE	0.479*	0.495*	0.436*	0.485*	0.499*	0.371*
SF-MH	0.235*	0.196**	0.189**	0.194**	0.228*	0.110
SF	0.490*	0.560*	0.481*	0.544*	0.530*	0.281*

*P < 0.01, **P < 0.05 (Spearman correlation analysis was used). T-PRWHE: Turkish version of the Patient Rated Wrist/Hand Evaluation; PRWHE-P: pain subscale of the T-PRWHE; PRWHE-SF: specific functional subscale of the T-PRWHE; PRWHE-UF: usual functional subscale of the T-PRWHE; PRWHE-F: functional subscale of the T-PRWHE; PRWHE-A: appearance subscale of the T-PRWHE; DASH-S: symptom subscale of the Disabilities of the Arm, Shoulder, and Hand Scale; DASH-W: work subscale of the DASH; SF-PF: physical functioning subscale of the 36-Item Short-Form Health Survey (SF-36); RP: role physical; BP: bodily pain; GH: general health; VT: vitality; SF: social functioning; RE: role emotional; MH: mental health.

The Kaiser–Meyer–Olkin (KMO) value was calculated for factor analysis of the principal components. The KMO value was found to be 0.91, exceeding the recommended value of 0.6 [26]. Principal component analysis revealed the presence of three factors: the first factor, specific function, explained 47% of the variance; the second factor, pain, explained 12% of the variance; and the third factor, usual function, explained 7% of the variance. Each item was represented in factor analysis and the results were parallel to the original criterion factor analysis. Factor analysis is summarized in Table 5.

**Table 5:** Component matrix of factor analysis for T-PRWHE.

Items		Componentomponen	
	1	2	3
P1	0.183	0.654	0.334
P2	0.075	0.758	0.217
P3	0.486	0.579	0.043
P4	0.089	0.710	–0.048
P5	0.216	0.724	0.237
SF1	0.748	0.346	0.205
SF2	0.814	0.305	0.111
SF3	0.793	–0.010	0.394
SF4	0.800	0.257	0.199
SF5	0.728	0.218	0.241
SF6	0.680	–0.018	0.353
UF1	0.535	0.081	0.716
UF2	0.535	0.257	0.601
UF3	0.259	0.145	0.723
UF4	0.146	0.252	0.747
PRWHE-P: Pain scale of the Turkish version of the Patient Rated			
Wrist/Hand Evaluation (T-PRWHE); PRWHE-SF: specific functional subscale of the T			
PRWHE; PRWHE-UF: usual functional subscale of the T-PRWHE.			

Baseline and follow-up PRWHE-A section scores are presented in Table 6.

**Table 6:** PRWHE-A values at baseline and at 3 months of follow-up.

Follow-up	PRWHE-A¥	Baseline			Total	Pǂ	Not important at all	A little important	Very important
Follow-up	Not important at all	25 (100%)	46 (79.3%)	60 (72.3%)	131 (78.9%)	<0.001*
	A little important	-	11 (19.0%)	16 (19.3%)	27 (16.3%)	
	Very important	-	1 (1.7%)	7 (8.4%)	8 (4.8%)	
Total		25 (15.1%)	58 (34.9%)	83 (50.0%)	166 (100%)	

T-PRWHE: Turkish version of the Patient Rated Wrist/Hand Evaluation. ¥ PRWHE-A: Appearance scale of the T-PRWHE. Data are expressed as number (%). ǂ Marginal homogeneity test was used. *P < 0.05 is considered statistically significant.

## 4. Discussion 

The PRWHE questionnaire is used to evaluate the pain and disability level in the wrist and hand of the patient, determine treatment targets, and follow the pain and functional status after rehabilitation and surgical treatment [15]. Its reliability and validity have been tested and it has been used in various populations as it is short and easy to administer [14–16].

In the case of the cross-cultural compliance of the PRWHE questionnaire from English to Turkish, both the forward and back translations did not indicate serious inconsistency. Since “pound” is not commonly used in Turkey as a measurement unit, the expression was converted to “kilogram”. 

The CA coefficient was used for internal consistency in the reliability analysis. Although the CA coefficients obtained in the present study were lower than in the original scale [4,5], they were over 0.70, the acceptable limit [27,28]. The reason why the CA coefficient was lower than in some other studies may be the inclusion of patients with various diagnoses. Hemelaers et al. evaluated only patients with distal radius fracture and found a CA coefficient of 0.89 for total PRWE score in the study that they conducted in the German population [29]. Moreover, the social differences in pain perception and evaluation may explain why the CA coefficient for PRWHE-P is lower in our study. Similar to the results of our study, the CA coefficient of the PRWE-P was found to be lower than the other subscales in the validity and reliability studies of the Chinese and German versions of PRWE questionnaire [8,29]. The test-retest reliability of the questionnaire was evaluated with ICCs. The ICC value was found to be over 0.90 for the T-PRWHE and its subscales and perfect test-retest reliability was obtained. 

Validity is the ability of an instrument to measure what it is intended to measure [27]. The statistical relationship between T-PRWHE and DASH and also T-PRWHE and SF-36 scores was assessed for the evaluation of criterion-related validity. The reason why these questionnaires were used is that their Turkish validity and reliability have been shown previously and they have been used as the gold standard in previous studies [10,12,16,29].

There was no relationship between the DASH-W and pain subgroup of the T-PRWHE, but there was a significant relationship between DASH and other subgroups of T-PRWHE. This may be due to the fact that the questions in the DASH-W are related to functionality and are inadequate in the evaluation of pain. Besides this, the correlation between subgroups of the SF-36 and T-PRWHE were found to be weak, consistent with the literature [8,12,29]. The reason for this is that the PRWHE questionnaire measures health-related quality of life only in relation to the single body region, while the SF-36 is a general assessment.

Basic components factor analysis was performed to determine the structural validity of the T-PRWHE questionnaire. Three factors were found to be different than in the original scale. The values for the questions in the PRWHE-UF subscale were high, except for the first two questions. The first two questions in the PRWHE-UF, which is the third factor, contribute to the PRWHE-SF subscale, which is the first factor. This was thought to stem from both the questions being similar to those in the PRWHE-SF and the lack of determinant questions for daily activities. The KMO test result was 0.91 and the Bartlett test result was significant at the P < 0.0001 level in our study. The KMO test value showed the sample size to be sufficient for factor analysis while the Bartlett test results showed the scale to be appropriate for factor analysis. 

Internal consistency, criterion-related correlation coefficients, and factor analysis were used to collect evidence for the reliability and validity of the T-PRWHE questionnaire. Results obtained as a result of these methods presented important information regarding the reliability and validity of the scale.

Rehabilitation and surgical treatment interventions directed at the upper extremities aim to decrease the pain and increase function. Appearance is not considered a component of disability. However, appearance is an important result from the patient’s point of view. The PRWHE also includes questions on appearance [5]. In this study, 141 patients claimed that they felt uncomfortable with the appearance of their hand by various degrees at baseline, but this number had decreased to 5 at follow-up. This showed that the PRWHE is an important tool for following the change in appearance. It can also be assumed that appearance is important for most patients and parallel to the improvement in other parameters.

An important disadvantage of the PRWHE questionnaire is that most of the questions included in the functional section are related to the dominant hand of the patient. Some patients answered the questions related to ‘cutting meat with a knife by using my aching hand’ as ‘I use only my right hand for this task’. When we asked the patient to respond to the question considering that he/she performs this task with the dominant hand, the answer given possibly did not reflect reality.

The heterogeneity of patient distribution can be considered as an important limitation of this study. Another limitation was that most of the patients were female and at an advanced age. Items such as ‘carrying a 5 kg object with my aching hand’ and ‘cutting meat with a knife by using my aching hand’ included in the specific function subscale may not be appropriate in the evaluation of this patient group as these activities require too much strength. 

We conclude that the T-PRWHE is a valid and reliable tool for the assessment of pain and function in Turkish patients with injuries involving the wrist and hand. We believe that the use of this questionnaire for self-assessment by patients with wrist and hand problems will contribute to better outcomes for this group.
